# Prognostic and Immunological Role of THBS2 in Colorectal cancer

**DOI:** 10.1155/2021/1124985

**Published:** 2021-08-21

**Authors:** Bin Deng, Xiu-Ping Liu, Xiong Wang

**Affiliations:** ^1^Department of Critical Care Medicine, Zhongnan Hospital of Wuhan University, Wuhan, China; ^2^Department of Clinical Laboratory, Hangzhou TCM Hospital Affiliated to Zhejiang Chinese Medical University, Hangzhou, China; ^3^Department of Laboratory Medicine, Tongji Hospital, Tongji Medical College, Huazhong University of Science and Technology, Wuhan, China

## Abstract

**Objective:**

Thrombospondin 2 (THBS2) acts as oncogenic or tumor suppressive gene in diverse cancers. Here we studied the prognostic and immunological role of THBS2 in colorectal cancer (CRC) using bioinformatic analysis.

**Methods:**

The genetic and protein expression of THBS2 in CRC were explored across several databases, including ONCOMINE, GEPIA2, TIMER 2.0, UALCAN and HPA databases. Correlation between THBS2 expression and clinical features in CRC was assessed using UALCAN tool. Prognostic analysis was performed using GEPIA2 and PrognoScan. Immune infiltration correlation with THBS2 in CRC was investigated with TIMER 2.0 and TISIDB. THBS2 binding and correlated genes were analyzed using String, GEPIA2, and TIMER 2.0.

**Results:**

THBS2 was significantly higher in CRC across multiple databases. Age and histological subtype were correlated with THBS2 level. High THBS2 expression correlated with short overall and disease-free survival. THBS2 expression was positively correlated with immune infiltrates in CRC. Moreover, extracellular matrix structural constituent and organization, PI3K-Akt pathway, were involved in the functional mechanisms of THBS2.

**Conclusions:**

THBS2 correlates with poor prognosis and immune infiltration in CRC. THBS2 may act as a prognostic and immunological biomarker for CRC.

## 1. Introduction

Colorectal cancer (CRC) is one of the most frequent lethal malignant cancers of the digestive tract worldwide [1]. Metastatic CRC usually develops from benign tumor over time, and the prevalence of CRC is still increasing. Patients with CRC are diagnosed at advanced stages, although remarkable advances in the diagnosis and treatment of CRC have achieved. The 5-year overall survival (OS) rate of metastatic CRC is less than 15% [2]. Diagnosis at early stage could increase the 5-year survival rate. Tumor microenvironment (TME) plays critical roles in tumor development involving the interaction between cancer cells, tumor infiltrating immune cells, and their supporting cells. Therefore, identifying biomarkers for CRC diagnosis and prognosis may be helpful in the diagnosis or treatment of CRC.

Thrombospondin 2 (THBS2) is one of the matricellular Ca^2+^-binding glycoproteins family members, released from various types of cells, and exerts its biological functions including angiogenesis, cell adhesion, cytoskeletal organization, apoptosis, and cell motility by binding with cell surface receptors, extracellular matrix proteins, and growth factors [3, 4]. THBS2 was highly expressed in different cancers including CRC [5]. THBS2 levels were correlated with favorable prognosis of gastric cancer [6]. THBS2 played a double-edged role in adenocarcinoma through oncogenic and anti-angiogenic function [7]. Tian et al. reported that THBS2 was significantly correlated to poor prognosis, lymph node metastasis, TNM stages but not gender and age of CRC in a small cohort with 73 patients [8].

In this study, we investigate gene expression, survival status, and immune infiltration correlation with THBS2 in CRC across multiple databases, to reveal the potential mechanisms of THBS2 in the pathogenesis and prognosis of CRC.

## 2. Materials and Methods

### 2.1. ONCOMINE

ONCOMINE database, https://www.oncomine.org/, was used to examine the expression of THBS2 in different cancers including 86,733 samples from 715 datasets. The threshold of fold change and P value and were defined as 2 and 1E-4, respectively.

### 2.2. GEPIA2

GEPIA2, http://gepia2.cancer-pku.cn/, integrates data from the TCGA and GTEx projects by standard processing pipelines [9]. The pan-cancer analysis of THBS2 expression, correlation analysis between THBS2 and correlated genes, OS, disease free survival (DFS) were performed using GEPIA2.

### 2.3. UALCAN

UALCAN, http://ualcan.path.uab.edu/, is widely used for analyzing OMICS data in cancer [10]. The mRNA and protein levels of THBS2, correlation with clinicopathological parameters were analyzed using UALCAN.

### 2.4. Human Protein Atlas

The Human Protein Atlas (HPA), https://www.proteinatlas.org/, is an online database for proteome analysis based on 26,941 antibodies for 17,165 proteins, and also contains transcriptome data from more than 8,000 patients [11]. We obtained immunohistochemical staining images of THBS2 in healthy and CRC tissues.

### 2.5. Timer 2.0

The TIMER 2.0, http://timer.cistrome.org/, is an online website used to investigate the pan-cancer analysis of gene expression or correlation, and immune infiltration [12]. In the present study, the correlation between THBS2 and immune infiltration of B cell, CD4+ T cell, CD8+ T cell, macrophage, neutrophil, and dendritic cells in CRC.

### 2.6. PrognoScan

PrognoScan, http://dna00.bio.kyutech.ac.jp/PrognoScan/, was used for survival analysis [13]. GSE17536 and GSE14333 two datasets were selected to study the correlation between THBS2 expression and the DFS in CRC.

### 2.7. TISIDB

TISIDB, http://cis.hku.hk/TISIDB/, is used to seek the interaction between cancer and immune system [14]. The correlation between THBS2 expression and 28 tumor infiltrating lymphocytes (TILs) across several cancers was achieved using “lymphocyte” module.

### 2.8. Enrichment Analysis

Experimentally determined protein-protein interaction (PPI) network of THBS2 was constructed using STRING (https://string-db.org), and analyzed with Cytoscape. Gene Oncology (GO) analysis, including biological processes (BP), molecular functions (MF), and cellular component (CC), and the Kyoto Encyclopedia of Genes and Genomes (KEGG) pathway enrichment were analyzed using the R language software [R-3.6.0] with the “ggplot2” R package.

## 3. Results

### 3.1. Transcriptional Level of THBS2 in CRC Patients

**Figure 1:**
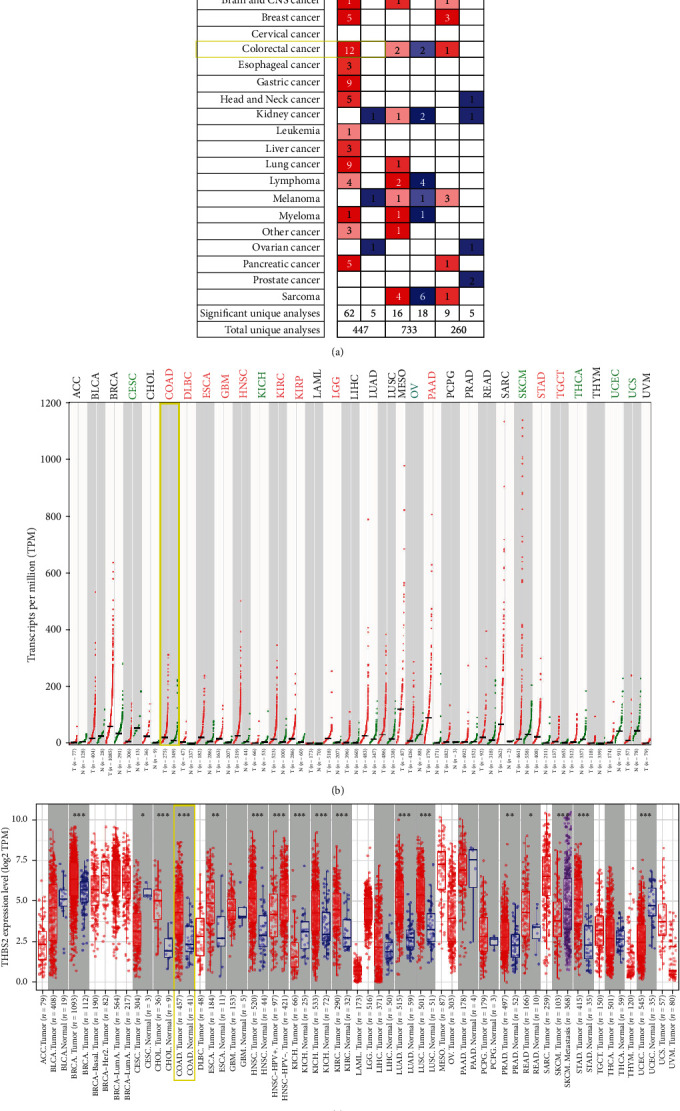
Pan-cancer analysis of THBS2 expression. (a) Pan-cancer analysis of THBS2 expression in ONCOMINE database. The number in each cell represented the number of datasets. (b) Pan-cancer analysis of THBS2 expression in GEPIA2 database. Tumor abbreviations in red color meant increased expression of THBS2 in tumors, while green color represented decreased expression of THBS2 in tumors. (c) Pan-cancer analysis of THBS2 expression in TIMER2.0 database. Colorectal cancer was labelled in yellow box. ∗P < 0.05, ∗∗P < 0.01, ∗∗∗P < 0.001.

The pan-cancer analysis of THBS2 in different cancers was analyzed in the ONCOMINE database. THBS2 expression was higher in several cancers, including breast, colorectal, esophageal, gastric, liver, lung, and pancreatic cancers (Figure 1(a)). Similar results were obtained from GEPIA2 (Figure 1(b)) and TIMER 2.0 (Figure 1(c)) databases. All these data suggest that THBS2 was highly expressed in CRC.

### 3.2. Correlation between THBS2 Expression and Clinical Features

**Figure 2:**
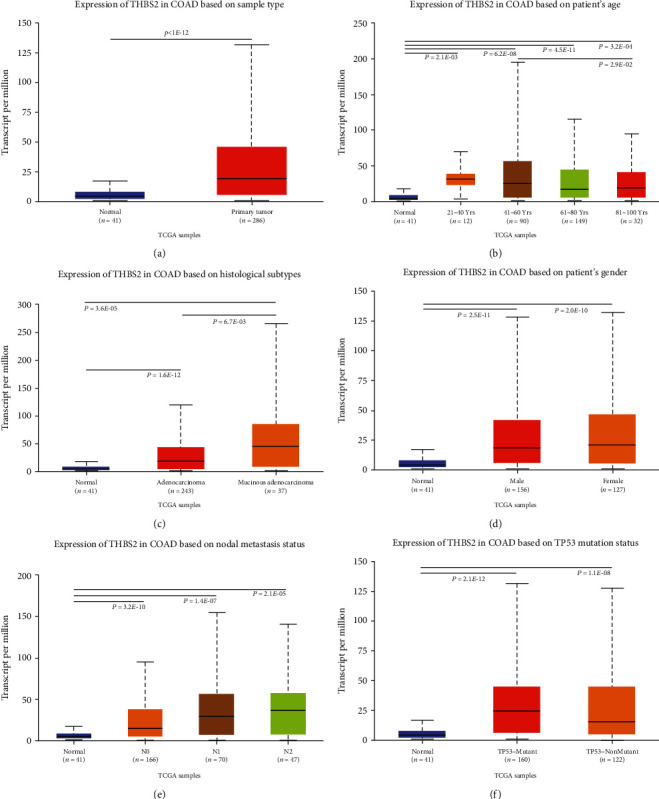
Box plot evaluating THBS2 expression of patients with CRC in different clinical characteristics. (a) Overall expression. (b) Age. (c) Histological subtype; (d) Gender; (e) Node metastasis; (f) TP53 mutation. All these data were analyzed using UALCAN database.

The expression of THBS2 was further found to be up-regulated in CRC using UALCAN database (Figure 2(a)). The correlation between THBS2 expression and clinical features in CRC patients was analyzed using UALCAN database according to age (Figure 2(b)), histological subtype (Figure 2(c)), gender (Figure 2(D)), node metastasis (Figure 2(e)), and TP53 mutation status (Figure 2(f)). THBS2 expression was only correlated with age and histological subtype in CRC.

### 3.3. Protein Expression of THBS2 in CRC

**Figure 3:**
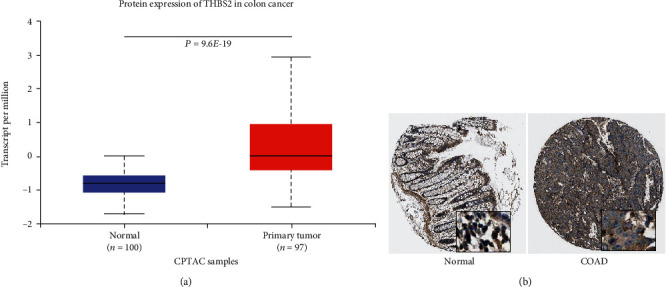
Protein levels of THBS2 in CRC. (a) Protein expression of THBS2 in CRC from UALCAN database. (b) Immunohistochemistry of THBS2 in CRC from HPA database.

The protein expression of THBS2 was studied in UALCAN (Figure 3(a)) and HPA (Figure 3(b)) databases. These results showed increased THBS2 protein levels in CRC, similar with its mRNA levels.

### 3.4. Prognostic Value of THBS2

**Figure 4:**
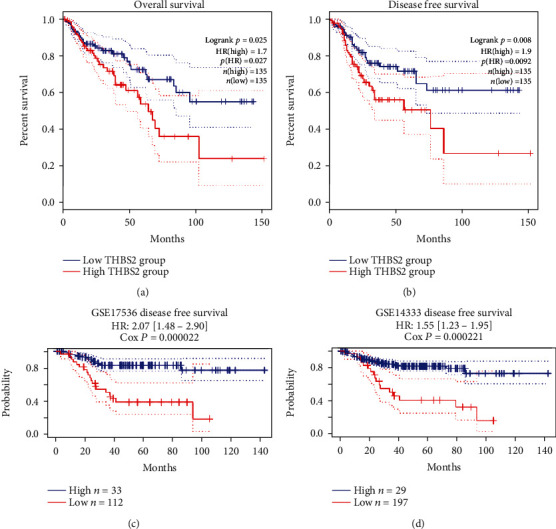
Kaplan-Meier curve for patients with CRC according to HTBS2 expression. (a) Kaplan-Meier curve for OS in CRC using dataset in GEPIA 2. (b) Kaplan-Meier curve for DFS in CRC using dataset in GEPIA 2. (c) Kaplan-Meier curve for DFS in CRC using dataset in GSE17536. (d) Kaplan-Meier curve for DFS in CRC using dataset in GSE14333.

We analyzed the relationship between THBS2 expression and the survival time of CRC patients in GEPIA2 and two GEO datasets. The result from GEPIA2 revealed that high level of THBS2 correlated with poor OS and DFS in CRC (Figure 4(a), 4(b)). GSE17536 and GSE14333 two datasets were selected to explore the relationship between THBS2 expression and DFS in CRC using PrognoScan. Both datasets showed similar results with GEPIA2 (Figure 4(c), 4(d)). High level of THBS2 was significantly correlated with poor prognosis of CRC and may be a useful biomarker to predict CRC survival rate.

### 3.5. Correlations between THBS2 Expression and Immune Infiltration

**Figure 5:**
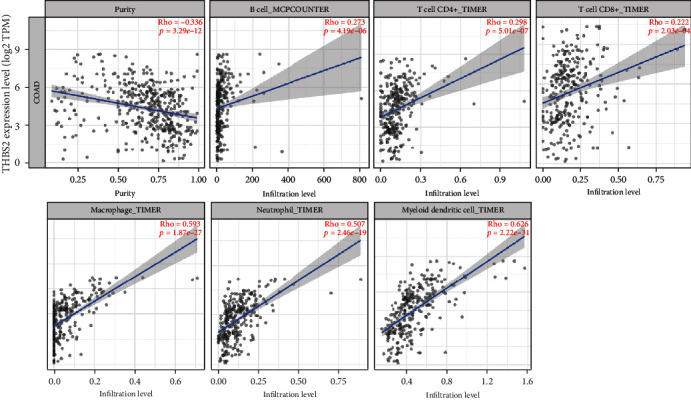
Correlation of THBS2 expression with immune infiltration in CRC. (A) THBS2 expression did not correlate with tumor purity and showed significant positive correlation with infiltrating levels of B cell, CD4+ T cell, CD8+ T cell, macrophage, neutrophil, and dendritic cells.

The association between immune infiltration and THBS2 expression was assessed by calculating the coefficient of THBS2 expression and immune infiltration using TIMER 2.0 database. The results indicated that THBS2 expression was significantly correlated with immune infiltration of B cells, CD4+ T cells, CD8+ T cells, neutrophils, macrophages, and dendritic cells (Figure 5).

**Figure 6:**
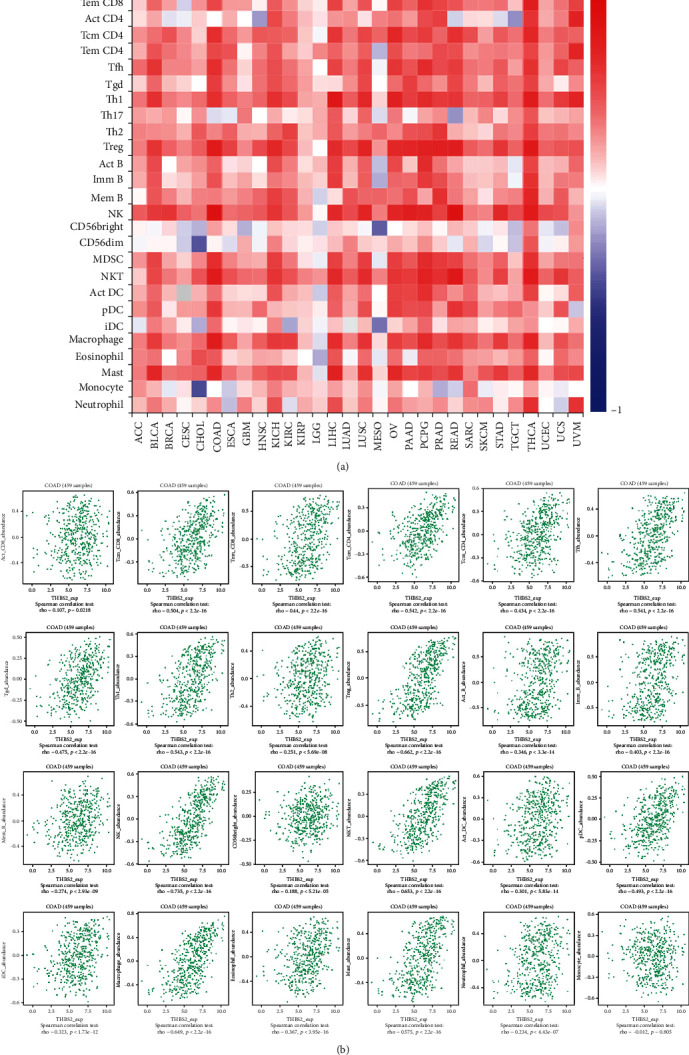
Correlation analysis of THBS2 expression with TILs in multiple cancers using TISIDB database. (a) Pan-cancer landscape of relationship between THBS2 expression and 28 TILs. (b) Detailed correlation between THBS2 expression and TILs in CRC.

The relationship between THBS2 expression and abundance of 28 TILs was investigated in TISIDB database. THBS2 was positively correlated with TILs in most cancers (Figure 6(a)). Specifically, in CRC, THBS2 expression was significantly correlated with 23 of 28 TILs (Figure 6(b)).

### 3.6. Enrichment Analysis of THBS2-Related Partners

**Figure 7:**
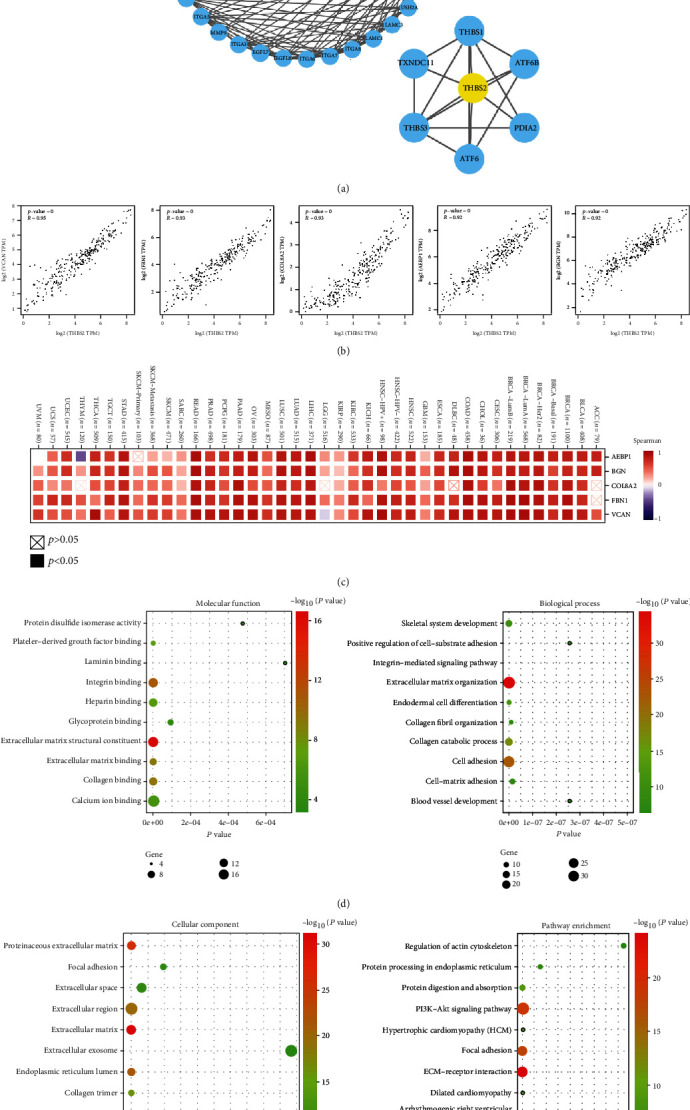
THBS2-related gene enrichment analysis. (a) Experimentally determined THBS2-binding proteins analyzed with STRING. (b) The top 100 THBS2-correlated genes in TCGA were obtained from GEPIA 2, and the expression correlation between THBS2 and top 5 genes were performed, including VACM, FBN1, COL8A2, AEBP1, and BGN. (c) The corresponding heat map in CRC was displayed. Based on the THBS2-binding and correlated genes, GO (d) and KEGG pathway (e) analysis was performed.

To study the potential molecular mechanisms of THBS2 in CRC tumorigenesis, THBS2-binding proteins and THBS2 correlated genes were screened using STRING and GEPIA2. A total of 38 THBS2-binding proteins were identified using STRING with experimental evidence. Moreover, the MCODE app was applied to select different modes. The mode included THBS2 was shown. THBS2 interacted with THBS1, THBS3, ATF6, ATF6B, PDIA2, and TXNDC11 in this mode (Figure 7(a)). The top 100 THBS2 correlated genes were achieved with GEPIA2 database. THBS2 expression was positively correlated with VACM, FBN1, COL8A2, AEBP1, and BGN (Figure 7(b)). The corresponding heat map also showed positive correlation between THBS2 and the top 5 genes in the several cancer types (Figure 7(c)).

The two datasets were combined to perform GO and KEGG enrichment analysis. The GO analysis suggested correlation with extracellular matrix structural constituent and organization (Figure 6(d)). KEGG data showed that ECM-receptor interaction and PI3K-Akt pathways were involved in the functional mechanisms of THBS2 (Figure 6(e)).

## 4. Discussion

Genetic and epigenetic processes play important roles in the initiation and progression of CRC [15].

THBS2 is a member of secreted calcium-binding glycoproteins mediating cell-cell and cell-matrix interactions. THBS2 is recognized as an ECM-modifying enzyme and functions as a suppressor of tumor growth and angiogenesis by interacting with matrix serine proteases and MMPs in numerous cancers [16]. On the other hand, the oncogenic role of THBS2 have also been observed [4, 7]. Here, we comprehensively examined the genetic and protein expression, prognostic, and immunological role of THBS2 in CRC based on datasets from TCGA, CPTAC and GEO databases.

The mRNA expression profile of THBS2 was obtained from ONCOMINE, GEPIA2, UALCAN, and TIMER 2.0 databases. Significant upregulations in cancer tissues was found across various cancer subtypes including breast, esophageal, colorectal, gastric, lung, liver, and pancreatic cancers, and decreased expression was only found in melanoma, kidney, and ovarian cancers as shown in ONCOMINE database. Similarly, protein levels of THBS2 were also decreased in CRC. Moreover, THBS2 expression was correlated with some clinicopathological parameters including patients' age and histological subtype. The KM-plots were obtained for the OS and DFS showed that high THBS2 expression correlated with poor prognosis of CRC in both GEPIA2 database and two GEO datasets.

The infiltrating immune cells in TME account for a large proportion, and immune infiltration into the TME prevent tumor cells from being killed [17]. Immunotherapy targeting interactions between tumor cells and immune cells have been used to create antitumoral immune responses by reactivating adaptive and innate immune systems [18]. In this study, THBS2 expression did not correlate with tumor purity and showed significant positive correlation with infiltrating levels of B cell, CD4+ T cell, CD8+ T cell, macrophage, neutrophil, and dendritic cells in TIMER 2.0 database. Moreover, A pan-cancer correlation analysis between THBS2 and 28 TILs showed positive correlation across multiple cancers. THBS2 was positively correlated with 23 of 28 TILs in CRC.

The co-expression and correlation of the top 5 positively correlated genes with THBS2 were depicted on the heat map (Figure 7(c)), including VACM, FBN1, COL8A2, AEBP1, and BGN. These genes showed positive correlation with THBS2 across most cancer types in TIMER 2.0 database. Collectively, THBS2 gene along with its associated VACM, FBN1, COL8A2, AEBP1, and BGN might act as prognostic biomarkers for CRC. The PPI networks indicate THBS1, THBS3, ATF6, ATF6B, PDIA2, and TXNDC11 as the most essential protein networks connected to the prognosis of CRC (Figure 7(a)). The GO analysis suggested that most of these genes are linked to extracellular matrix structural constituent and organization. KEGG data showed that ECM-receptor interaction and PI3K-Akt pathways were involved in the functional mechanisms of THBS2. Ao et al. reported that THBS2 silencing suppressed gastric cancer cell proliferation, migration, and invasion through the PI3K-Akt pathway [4].

In summary, we analyzed the prognostic landscape of THBS2 in CRC across multiple databases, explored the correlation between THBS2 expression and immune infiltration, and finally identified the THBS2 binding proteins and correlated genes in cancer. The findings indicate that THBS2 correlates with poor prognosis of CRC patients partially through its interaction with infiltrating immune cells and correlated genes in CRC.

## Data Availability

The data used to support the findings of this study are included within the article.

## Abbreviations

CRC: Colorectal cancer
DFS: Disease free survival
OS: Overall survival
TME: Tumor microenvironment
THBS2: Thrombospondin 2
TIL: Tumor infiltrating lymphocyte
ECM: Extracellular matrix
PPI: Protein-protein interaction
KEGG: Kyoto encyclopedia of genes and genomes
GO: Gene oncology.
